# Development of an electrotransformation protocol for genetic manipulation of *Clostridium pasteurianum*

**DOI:** 10.1186/1754-6834-6-50

**Published:** 2013-04-09

**Authors:** Michael E Pyne, Murray Moo-Young, Duane A Chung, C Perry Chou

**Affiliations:** 1 Department of Chemical Engineering, University of Waterloo, 200 University Avenue West, Waterloo, ON, N2L 3G1, Canada; 2 Centurion Biofuels, Corp., Rm. 5113 Michael G. DeGroote Centre for Learning and Discovery, 1280 Main Street West, Hamilton, ON, L8S 4K1, Canada

**Keywords:** Biofuels, Butanol, Biobutanol, *Clostridium pasteurianum*, Electroporation, Genetic engineering, Glycerol, Methylation, Restriction, Transformation

## Abstract

**Background:**

Reducing the production cost of, and increasing revenues from, industrial biofuels will greatly facilitate their proliferation and co-integration with fossil fuels. The cost of feedstock is the largest cost in most fermentation bioprocesses and therefore represents an important target for cost reduction. Meanwhile, the biorefinery concept advocates revenue growth through complete utilization of by-products generated during biofuel production. Taken together, the production of biofuels from low-cost crude glycerol, available in oversupply as a by-product of bioethanol production, in the form of thin stillage, and biodiesel production, embodies a remarkable opportunity to advance affordable biofuel development. However, few bacterial species possess the natural capacity to convert glycerol as a sole source of carbon and energy into value-added bioproducts. Of particular interest is the anaerobe *Clostridium pasteurianum*, the only microorganism known to convert glycerol alone directly into butanol, which currently holds immense promise as a high-energy biofuel and bulk chemical. Unfortunately, genetic and metabolic engineering of *C. pasteurianum* has been fundamentally impeded due to lack of an efficient method for deoxyribonucleic acid (DNA) transfer.

**Results:**

This work reports the development of an electrotransformation protocol permitting high-level DNA transfer to *C. pasteurianum* ATCC 6013 together with accompanying selection markers and vector components. The CpaAI restriction-modification system was found to be a major barrier to DNA delivery into *C. pasteurianum* which we overcame by *in vivo* methylation of the recognition site (5’-CGCG-3’) using the M.FnuDII methyltransferase. With proper selection of the replication origin and antibiotic-resistance marker, we initially electroporated methylated DNA into *C. pasteurianum* at a low efficiency of 2.4 × 10^1^ transformants μg^-1^ DNA by utilizing conditions common to other clostridial electroporations. Systematic investigation of various parameters involved in the cell growth, washing and pulse delivery, and outgrowth phases of the electrotransformation procedure significantly elevated the electrotransformation efficiency, up to 7.5 × 10^4^ transformants μg^-1^ DNA, an increase of approximately three order of magnitude. Key factors affecting the electrotransformation efficiency include cell-wall-weakening using glycine, ethanol-mediated membrane solubilization, field strength of the electric pulse, and sucrose osmoprotection.

**Conclusions:**

*C. pasteurianum* ATCC 6013 can be electrotransformed at a high efficiency using appropriately methylated plasmid DNA. The electrotransformation method and tools reported here should promote extensive genetic manipulation and metabolic engineering of this biotechnologically important bacterium.

## Background

As a promising high-energy biofuel and bulk chemical, butanol is produced fermentatively by select members of the genus *Clostridium*[1]. Recent efforts to reinstate an economical fermentation-based butanol industry able to rival current petrochemical production routes have thus far proven uncompetitive [2]. Restrictive feedstock cost, accounting for up to 80% of total biobutanol production [3,4], is often regarded as the chief factor limiting industrialization. Advantageously, tremendous expansion experienced by the global biodiesel industry in recent years has led to a significant oversupply of crude glycerol, which is generated as a waste stream representing approximately 10% (w/w) of the unpurified biodiesel product [5-7]. In analogy to the biodiesel industry, immense rise in global bioethanol production has led to the accumulation of thin stillage, the aqueous fraction of whole stillage and an inexpensive waste product possessing a high proportion of unpurified, fermentable glycerol [8]. Accordingly, glycerol has become a highly attractive fermentable substrate due to its low cost and high degree of reduction.

Despite the notoriously wide substrate utilization range exhibited by the clostridia for butanol production, few species are able to grow on glycerol as a sole carbon and energy source. The prototypical industrial butanol producers, *Clostridium acetobutylicum* and *C. beijerinckii*, can only metabolize glycerol if the growth medium is supplemented with a less reduced carbon source, such as glucose [9], thus devaluing the economic advantages afforded by waste glycerol feedstocks. Though *C. butyricum* can ferment glycerol without glucose supplementation, it does not express a functional butanol biosynthetic pathway [10]. Among the clostridia, *C. pasteurianum* appears to be the only species that combines the capacity for glycerol dissimilation with a highly active butanol-producing pathway [11]. Under proper conditions for cultivation of *C. pasteurianum* using glycerol, butanol, ethanol, and 1,3-propanediol dominate, while organic acids are produced in only trace amounts [12]. Numerous recent studies have investigated the fermentation of pure and crude glycerol [10,12-17], in addition to thin stillage [18], by *C. pasteurianum*.

Unlike the past industrial workhorses, *C. acetobutylicum* and *C. beijerinckii*, *C. pasteurianum* has garnered nominal attention as a potential host for the production of butanol. This is largely due to the current inability to transfer DNA to *C. pasteurianum*, in addition to lack of a genome sequence for this organism. Based on early genetic studies, it appears efforts were in place to conduct genetic manipulation of *C. pasteurianum*, since a method for producing and regenerating protoplasts was developed [19] and a Type-II restriction endonuclease was identified as a potential barrier to gene transfer [20]. Successful conjugation-based plasmid transfer to *C. pasteurianum* has also been documented [20], yet no protocol has been described, nor have any genetic mutants arisen from this work. Accordingly, no genetic tools are currently available for the manipulation of *C. pasteurianum*. Recent efforts to improve *C. pasteurianum* butanol production have focused on traditional and less favorable random chemical mutagenesis techniques [21,22]. In order to construct superior *C. pasteurianum* strains through rational genetic and metabolic engineering strategies, it is pertinent to develop methods to transfer foreign DNA to this microorganism.

In contrast to Gram-negative bacteria, Gram-positive cells possess an extensive exterior network of peptidoglycan which physically restricts passage of exogenous DNA into the cell. For this reason, electrotransformation of Gram-positive species is generally less efficient than Gram-negative strains [23]. To overcome the thick Gram-positive cell wall, mild or brief pretreatment using cell-wall-weakening agents, such as lysozyme, glycine, dl-threonine, or penicillin G, is commonly required to achieve optimal electrotransformation while maintaining sufficient cell viability [23,24]. Whereas some species of *Clostridium* can be electrotransformed without the use of cell-wall-weakening agents [25,26], others are electrotransformed at elevated levels when treated with such additives [27-29]. Poor electrotransformation efficiency of Gram-positive bacteria is further compounded within the clostridia due to the unusually high production of non-specific cell-wall-associated nucleases [26]. A number of highly-specific clostridial Type-II restriction endonucleases have also been identified [25,26,30], including CpaAI from *C. pasteurianum* ATCC 6013 [20], highlighting the importance of DNA protection via methylation of the transforming DNA. Unidentified restriction-modification systems are likely the underlying cause of electrotransformation recalcitrance that has been observed with certain species, such as *C. butyricum*[31]. In summary, development of electrotransformation within the clostridia entails investigation of cell-wall-weakening additives, inactivation or evasion of non-specific nucleases, and protection of foreign DNA against highly specific restriction endonucleases, in addition to examination of other common parameters involved in the cell growth, washing and pulse delivery, and recovery phases of the standard bacterial electrotransformation procedure [23,24].

As an entry point to allow genetic manipulation of *C. pasteurianum*, here we report the development of an electroporation-mediated transformation system for *C. pasteurianum* ATCC 6013. CpaAI was validated as a major restriction endonuclease attacking foreign DNA delivered into *C. pasteurianum* and this mechanistic limitation was resolved by *in vivo* methylation of the recognition site (5’-CGCG-3’) prior to electroporation. Methylation alone, however, did not result in high-level transfer of DNA into *C. pasteurianum*. Instead, through systematic investigation, we developed an efficient electrotransformation method that is dependent on weakening of the cell wall using glycine, ethanol-mediated membrane solubilization, a low electric field, and osmotic stabilization afforded by sucrose. In addition to the electroporation protocol, we also identified effective antibiotic selection systems and origins of replication capable of sustaining plasmids in *C. pasteurianum*. To our knowledge, this study presents the first demonstration of DNA transfer into *C. pasteurianum* with a high efficiency and opens an avenue for extensive genetic and metabolic engineering of *C. pasteurianum*.

## Results

### Protection of plasmid DNA from CpaAI restriction

**Figure 1:**
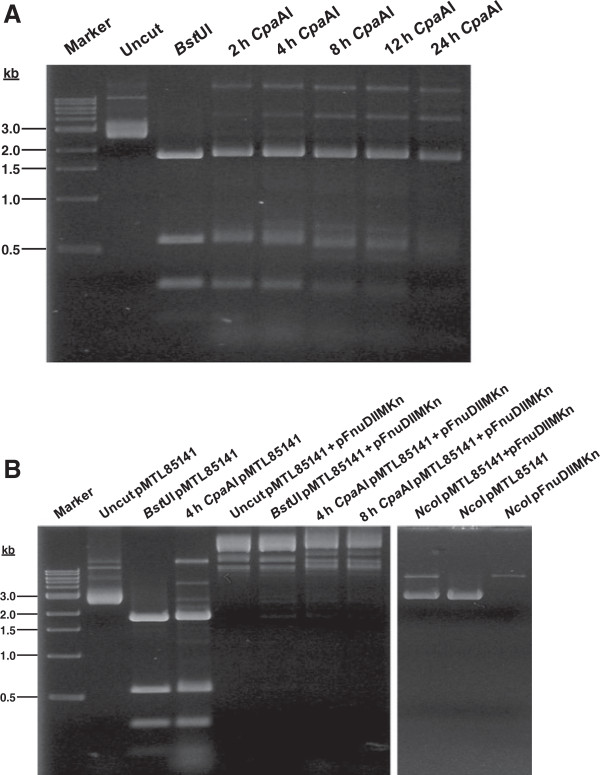
**M.FnuDII methyltransferase-mediated protection of pMTL85141 against CpaAI endonuclease. A.** Time course digestion of pMTL85141 using crude protoplast extracts possessing CpaAI restriction activity, resolved on a 2% agarose gel. Digestion reactions contained 1.0 μg pMTL85141 and 25% protoplast extract in a total volume of 20 μl 1× CpaAI custom buffer. For comparison, pMTL85141 is shown undigested and digested with BstUI, a commercial isoschizomer of CpaAI. Expected digestion products are 1785, 581, 270, 252, and 75 bp. **B.** M.FnuDII-mediated protection of pMTL85141 from CpaAI digestion (left panel). Protoplast extract digestions contained 1.0 μg pMTL85141 or pMTL85141+pFnuDIIMKn, the vector harboring the M.FnuDII methyltransferase gene, and 25% protoplast extract in a total volume of 20 μl 1× CpaAI custom buffer. pMTL85141 preparation in the presence of plasmid pFnuDIIMKn afforded protection of pMTL85141 from both BstUI and CpaAI restriction, as no digestion products could be detected. Methylation treatment resulted in the presence of high-molecular weight bands. Linearization of the high molecular weight bands by NcoI digestion (right panel) confirmed the presence of pMTL85141 (2,963 bp) and the methylating plasmid, pFnuDIIMKn (6,449 bp), at the correct sizes of the individual linearized vectors.

To develop a *C. pasteurianum* transformation protocol, we first assayed crude cell lysates for the presence of restriction-modification systems, which potently inhibit plasmid DNA transfer to bacteria. At least one Type-II restriction endonuclease, designated CpaAI with 5’-CGCG-3’ recognition and an isoschizomer of ThaI and FnuDII, has been previously identified in cell-free lysates of *C. pasteurianum* ATCC 6013 [20]. We initially prepared crude cell lysates through sonication of whole cells. As found in other species, such as *C. acetobutylicum*[26,32], lysates generated in this manner potently degraded all plasmid DNA substrates, presumably due to non-specific cell-wall-associated nucleases (data not shown). To overcome non-specific nuclease activity, we then aimed to assay CpaAI restriction activity using protoplast extracts, which allowed clear detection of CpaAI activity. Optimal digestion occurred between 2–4 hours incubation at 37°C and produced a restriction pattern identical to that of BstUI, a commercial isoschizomer of CpaAI (Figure 1A). Since all known BstUI isoschizomers catalogued in REBASE [33] are sensitive to methylation of both external cytosine residues within the 5’-CGCG-3’ recognition sequence, we next assessed the effect of external cytosine methylation by expression of the M.FnuDII methyltransferase (with 5’-^m^CGCG-3’ methylation site of both DNA strands) from plasmid pFnuDIIMKn. M.FnuDII methylation protected pMTL85141, an *E. coli-Clostridium* shuttle vector [34], from degradation by CpaAI and BstUI (Figure 1B). While unmethylated substrates were significantly restricted after 2 hours incubation at 37°C, M.FnuDII-methylated substrates were completely resistant to cleavage, even after 8 h. Note that methylated pMTL85141 plasmid preparations, which also contains the pFnuDIIMKn methylating plasmid, migrated at a different molecular weight than unmethylated plasmid preparations. However, when we linearized the double-plasmid preparation, in addition to preparations of the two individual plasmids, we observed no detectable changes in plasmid size or unexpected products (Figure 1B, right panel). *In vitro* methylation with commercial M.SssI (5’-^m^CG-3’ methylation site) and M.CviPI (5’-GmC-3’ methylation site) methyltransferases also protected plasmids from digestion by CpaAI in protoplast extracts and commercial BstUI (not shown).

### Initial electrotransformation of *C. pasteurianum*

To electrotransform *C. pasteurianum*, we employed a series of *E. coli*-*Clostridium* shuttle vectors which differ only in their Gram-positive origins of replication: pMTL82151 (pBP1 ori from *C. botulinum*); pMTL83151 (pCB102 ori from *C. butyricum*); pMTL84151 (pCD6 ori from *C. difficile*); and pMTL85141 (pIM13 ori from *Bacillus subtilis*) [34]. We utilized conditions common to clostridial electrotransformation procedures (Table 1) and M.FnuDII-methylated DNA. Of the four vectors tested, pMTL83151, pMTL84151, and pMTL85141 yielded colonies using thiamphenicol selection, corresponding to electrotransformation efficiencies of 0.7 × 10^1^, 0.3 × 10^1^, and 2.4 × 10^1^ transformants μg^-1^ DNA, respectively. Accordingly, pMTL85141 was selected as the vector used for all subsequent electrotransformation work. Importantly, no transformants were obtained with unmethylated plasmid, validating the necessity to protect transforming DNA against the endogenous CpaAI restriction endonuclease. Interestingly, while *in vivo* methylation was essential for transformation, we did not obtain transformants when pMTL85141 was methylated *in vitro* with M.SssI or M.CviPI methyltransferases, although both enzymes protect pMTL85141 from digestion by CpaAI. This result is unexpected, but is speculated upon later in this report.

**Table 1:** **Consensus clostridial electrotransformation conditions leading to initial low-level transformation of *C. pasteurianum***

**Electrotransformation parameter**	**Consensus for *Clostridium *species**	**Low-level transformation of *C. pasteurianum***	**Selected references**
*Cell growth*			
Growth medium	YTG or 2×YTG	2×YTG	[44,58,59]
Growth phase and OD_600_ at time of harvest	mid to late exponential phase (OD_600_ 0.5–0.9)	OD_600_ 0.6-0.8	[25]
*Washing and pulse delivery*			
Wash and electroporation buffer	5–7 mM sodium phosphate, pH 6.5-7.4, containing 270 mM sucrose and 1 mM MgCl_2_	5 mM sodium phosphate, pH 6.5, containing 270 mM sucrose and 1 mM MgCl_2_	[25,26,29]
Number of wash steps	1	1	[44,45,58]
Cuvette gap width	0.4 cm	0.4 cm	[29,45,46]
Volume of cells	600 μl	600 μl	[26]
Pulse parameters	2.0–2.5 kV; 25 μF; 200–800 Ω; 4–8 ms	2.0–2.5 kV; 25 μF; 200–800 Ω; 6–9 ms	[44,46]
*Outgrowth*			
Recovery and plating medium	YTG or 2×YTG	2×YTG	[58,59]
Transformation efficiency	Up to 10^6^ transformants μg^-1^ DNA	2.4 × 10^1^ transformants μg^-1^ pMTL85141	[35]

**Figure 2:**
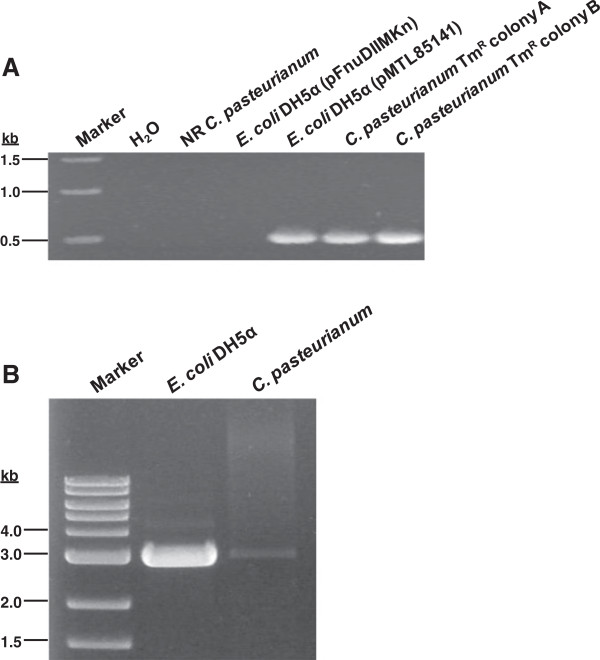
**Low-level electrotransformation of *C. pasteurianum*. A.** Colony PCR confirmation of pMTL85141 presence in *C. pasteurianum* transformants using primers pMTL.seq.S and pMTL.seq.AS. The expected product of 518 bp could only be amplified from *E. coli* transformed with pMTL85141 and from thiamphenicol-resistant *C. pasteurianum* colonies, and not from non-recombinant [66]*C. pasteurianum*. **B.** XhoI-linearized pMTL85141 plasmid prepared from *E. coli* DH5α and from a representative transformant of *C. pasteurianum* showing the expected plasmid size of 2,963 bp. Some undigested vector remains visible in the *E. coli* preparation.

To confirm the presence of pMTL85141 in transformed colonies, we screened thiamphenicol-resistant colonies for the presence of the *catP* resistance marker within pMTL85141 using colony PCR (Figure 2A). All of the colonies screened generated a single expected product of 518 bp. To further confirm the presence of plasmid and determine if rearrangements or recombinations occurred upon transfer to *C. pasteurianum*, plasmid pMTL85141was isolated and purified from thiamphenicol-resistant colonies and digested with XhoI. XhoI digestion of all plasmid preparations from *C. pasteurianum* yielded a single band on a 1.0% agarose gel, similar to the digestion of pMTL85141 prepared from *E. coli* DH5α (Figure 2B). The presence of the methyltransferase vector, pFnuDIIMKn, could not be detected in *C. pasteurianum* plasmid preparations.

### High-level electrotransformation of *C. pasteurianum*

An electrotransformation efficiency of 2.4 × 10^1^ transformants μg^-1^ DNA is very low relative to the efficiency of other clostridia (i.e., up to 10^6^ transformants μg^-1^ DNA [35]) and did not permit transfer of certain gene knockout vectors, as described later in this report. Therefore, we were prompted to develop a high-level electrotransformation protocol by systematically investigating the effects on electrotransformation efficiency of parameters throughout all phases of the electrotransformation procedure. Electrotransformation efficiencies reported below represent the average of at least two electrotransformation experiments using the same preparation of electrocompetent cells.

**Figure 3:**
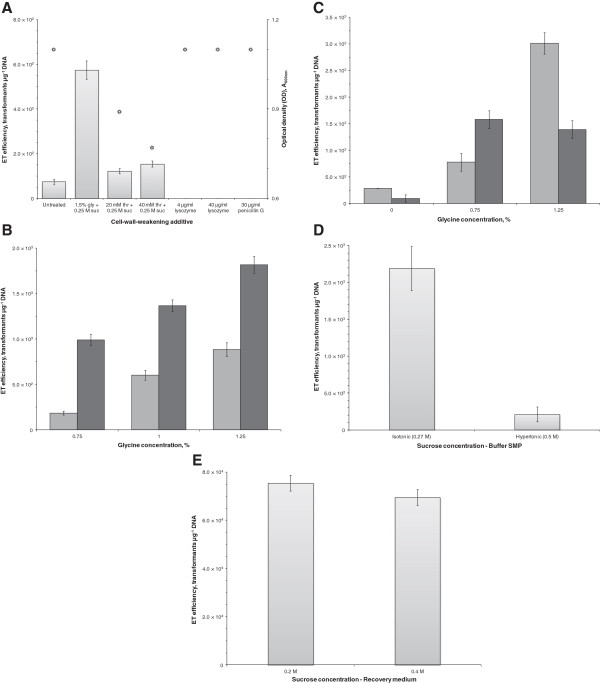
**Investigation of cell-wall-weakening and osmoprotection on electrotransformation of *C. pasteurianum*. A.** Investigation of cell-wall-weakening agents. Cells were grown to early exponential phase (OD_600_ 0.3-0.4) and glycine (gly) or dl-threonine (thr) was added along with 0.25 M sucrose (suc). For lysozyme and penicillin G treatments, additives were supplemented to buffer SMP prior to electroporation and incubated anaerobically at 37°C for 30 minutes. An untreated culture was included as a control. The OD_600_ of each culture at time of harvest is shown (**ο**). Pulse duration was unaffected between samples. **B.** Investigation of glycine and sucrose concentrations. Six cultures were grown to an OD_600_ of 0.4 and glycine was added to a final concentration of 0.75, 1.0, or 1.25% together with sucrose at 0.25 (light shading) or 0.4 M (dark shading). Growth was minimally affected between samples, as all cultures attained a final OD_600_ of 1.2-1.5. Pulse duration was unaffected between samples. **C.** Investigation of glycine concentration and duration of exposure. Two cultures were grown to an OD_600_ of 0.4 and glycine was added to a final concentration of 0.75 or 1.25% together with 0.4 M sucrose. An additional control culture was prepared without either glycine or sucrose supplementation. Cells were harvested, washed, and electroporated at either 2.5 (light shading) or 4 hours (dark shading) following supplementation with glycine and sucrose. Pulse duration was unaffected between samples. **D.** Effect of sucrose concentration within the wash and electroporation buffer. Cultures were washed and electroporated in SMP buffer containing either isotonic (0.27 M) or hypertonic sucrose (0.5 M). Pulse duration was unaffected between samples. **E.** Effect of sucrose concentration within the outgrowth medium. Cultures were electroporated and resuspended and grown in 2×YTG medium containing either 0.2 or 0.4 M sucrose.

**Figure 4:**
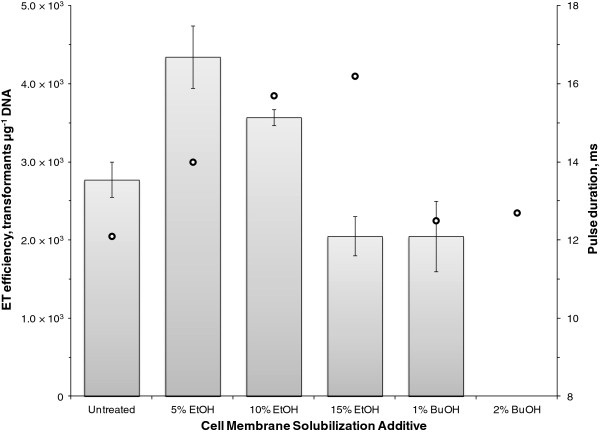
**Investigation of membrane permeabilization on the electrotransformation of *C. pasteurianum*.** Immediately prior to pulse delivery, cell-DNA suspensions were supplemented with 5, 10, or 15% ethanol (EtOH) or 1 or 2% butanol (BuOH). An untreated sample was included as a control. The time constant of each pulse is shown (**ο**). The sample treated with 2% butanol failed to grow during the allotted 16-hour recovery period following electroporation.

**Figure 5:**
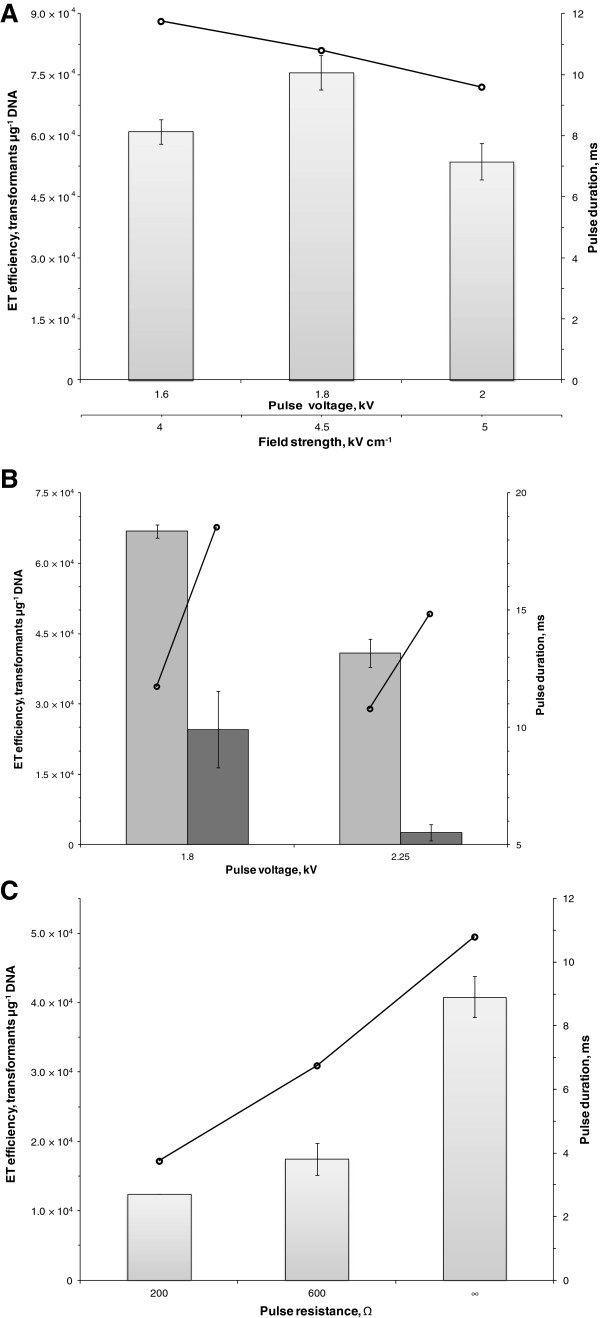
**Investigation of electric pulse parameters on the electrotransformation of *C. pasteurianum*. A.** Effect of pulse voltage (field strength). Electrotransformation efficiency was measured using electric pulses of 1.6, 1.8, or 2.0 kV, corresponding to field strengths of 4.0, 4.5, and 5.0 kV cm^-1^. The time constant of each pulse is shown (**ο**). **B.** Effect of pulse capacitance. Electrotransformation efficiency was measured at 25 (light shading) and 50 μF (dark shading) under voltages of 1.8 and 2.25 kV. The time constant of each pulse is shown (**ο**). **C.** Effect of pulse resistance. Electrotransformation efficiency was measured at 200, 600, and ∞ Ω at a voltage of 2.25 kV. The time constant of each pulse is shown (**ο**).

**Figure 6:**
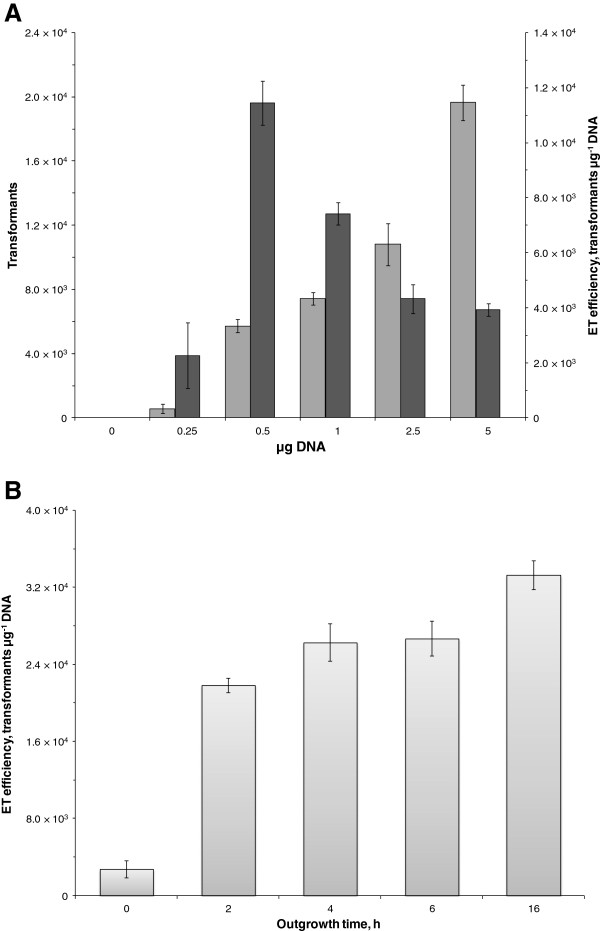
**Investigation of amount of DNA and outgrowth duration on the electrotransformation of *C. pasteurianum*. A.** Effect of plasmid DNA amount on total number of transformants and electrotransformation efficiency of *C. pasteurianum*. Separately, 0, 0.25, 0.5, 1.0, 2.5, and 5.0 μg of pMTL85141 were added to electrocompetent cells of *C. pasteurianum* and electroporated. Total number of thiamphenicol-resistant transformants (light shading) and electrotransformation efficiency (dark shading) were quantified. Pulse duration was unaffected between samples. Zero μg pMTL85141 failed to generate thiamphenicol-resistant transformants. **B.** Effect of post-electroporation incubation time. Cells were electroporated, transferred to 10 ml outgrowth medium containing 0.2 M sucrose, and incubated for 0, 2, 4, 6, or 16 hours prior to selective plating.

(i) **Cell-wall-weakening.** We first investigated the use of cell-wall-weakening agents due to their potential to greatly enhance electrotransformation by weakening of the Gram-positive cell wall [24]. A screening experiment was conducted to identify potential additives capable of enhancing electrotransformation of *C. pasteurianum*, including glycine, dl-threonine, lysozyme, and penicillin G (Figure 3A). Individually, we screened the effect of glycine and dl-threonine by supplying the additives in the presence of 0.25 M sucrose at the first signs of growth (OD_600_ of 0.3-0.4) because cultures failed to grow to sufficient cell densities if glycine or dl-threonine were present without sucrose supplementation or if the additives were present at the time of inoculation. Cell growth rate was slightly reduced in the presence of both glycine and dl-threonine. On the other hand, lysozyme and penicillin G were screened by addition at the wash stage in the wash and electroporation buffer, followed by incubation at 37°C for 30 minutes prior to electroporation. Additive concentrations were chosen based on previous electrotransformation studies with various species of Gram-positive bacteria [29,36-39]. Of the four additives screened, only glycine and dl-threonine improved the electrotransformation efficiency. The samples treated with 40 μg/ml lysozyme and 30 μg/ml penicillin G even failed to grow during the outgrowth period following electroporation, potentially due to cell lysis. Despite a slight inhibition on cell growth, more than 7-fold enhancement of electrotransformation efficiency was attained using 1.5% glycine, compared to the control experiment with no cell-wall-weakening agent. Supplementation of 20 and 40 mM dl-threonine provided approximately 1.6- and 2.1-fold increases, respectively, in electrotransformation efficiency. Although glycine and dl-threonine have different mechanisms of cell wall disruption, combining glycine and dl-threonine treatments did not lead to a synergistic increase in electrotransformation efficiency.

As a result of the clear benefit of glycine on the electrotransformation efficiency, we set out to determine the optimum glycine regimen with respect to concentration and duration of exposure. This investigation was done concomitant with investigating the effect of sucrose on electrotransformation efficiency by providing osmoprotection during the various cell-wall-weakening glycine treatments. We tested glycine at 0.75, 1.0, and 1.25% in the presence of either 0.25 or 0.4 M sucrose, corresponding to nearly isotonic and hypertonic extracellular environments, respectively. The highest glycine concentration was selected as 1.25% to minimize growth inhibition, which becomes significant at concentrations equal to or greater than 1.5%. Increasing the sucrose concentration from 0.25 to 0.4 M led to a significant increase in electrotransformation efficiency under all glycine concentrations tested (Figure 3B). To examine the effect of the duration of glycine exposure on electrotransformation efficiency, cultures were incubated with 0, 0.75, or 1.25% glycine in the presence of 0.4 M sucrose starting at an OD_600_ of 0.4 for either 2.5 or 4.5 hours prior to washing and pulse delivery (Figure 3C). Maximum electrotransformation efficiency was attained by exposing cells to 1.25% glycine for 2.5 hours in the presence of 0.4 M sucrose, a 10.7-fold increase compared to the untreated control culture. Interestingly, lower glycine concentrations could be compensated for by increasing the duration of exposure. When using a glycine concentration of 0.75% in the growth medium, 4.5 hours rather than 2.5 hours of exposure generated a greater electrotransformation efficiency at this lower glycine concentration, although the absolute gain in electrotransformation efficiency was still lower than with 1.25% glycine.

(ii) **Osmoprotection.** We continued to investigate the effect of the osmoprotectant concentration on electrotransformation efficiency during the subsequent washing and electroporation phase and the outgrowth phase following electroporation. Cells grown in the presence of 1.25% glycine and 0.4 M sucrose were washed and electroporated in the common clostridial SMP buffer containing either 0.27 M (isotonic) or 0.5 M (hypertonic) sucrose (Figure 3D). SMP buffer outperformed other buffers tested, such as 10% PEG 8000, 15% glycerol, protoplast buffer with lysozyme omitted, and SMP buffer supplemented with 15% glycerol (data not shown). Hypertonic sucrose, which improved the electrotransformation efficiency when included during the growth phase, reduced electrotransformation efficiency by a factor of 10.3 when included at the washing and electroporation phase. Thus, 0.27 M sucrose was adopted as the optimum sucrose concentration in the wash and electroporation buffer.

To assess the effect of sucrose osmoprotection during cell recovery immediately following delivery of the electric pulse, cells were grown, made electrocompetent, pulsed, and resuspended in 10 ml 2×YTG containing either 0.2 or 0.4 M sucrose (Figure 3E). Similar to the washing and electroporation phase, hypertonic sucrose again reduced electrotransformation efficiency, although the effect was modest (a 1.1-fold decrease), and thus, 0.2 M was adopted as the optimum sucrose concentration in the outgrowth medium.

(iii) **Cell membrane solubilization.** After developing a regimen to weaken the exterior cell wall while supporting cell viability with sucrose osmoprotection, we next sought to enhance transfer of plasmid DNA to *C. pasteurianum* with the use of ethanol to solubilize the cell membrane, a strategy which has proved effective with some species of Gram-negative bacteria [40,41]. We also extended this approach to butanol, which elicits a more pronounced toxic effect on cells. To achieve maximum membrane solubilization without adversely affecting cell viability, we utilized concentrations near the toxicity threshold for many species of *Clostridium*, which were up to 15% (v/v) for ethanol and 2% (v/v) for butanol [42,43]. Five minutes prior to electroporation, ethanol or butanol was added directly to the cell-DNA suspension. Ethanol added at 5 and 10% provided a 1.6- and 1.3-fold respective increase in electrotransformation efficiency, compared to the control experiment with no ethanol treatment (Figure 4). Butanol, and ethanol at an elevated concentration of 15%, proved to be detrimental to electrotransformation. The 2% butanol sample grew extremely slowly during the outgrowth period following electroporation. The addition of ethanol increased the pulse time constant, which may have influenced electrotransformation efficiency (Figure 4). Butanol did not significantly affect the pulse time constant.

(iv) **Electric pulse parameters.** We investigated the effects of the electrical pulse with respect to voltage (i.e., field strength), capacitance, and resistance (Figure 5A-C). In an initial screening experiment, low voltages in the range of 1.8-2.0 kV generated significantly more transformants than voltages of 2.0-2.5 kV (data not shown), which are representative of most electrotransformation protocols using species of *Clostridium*[29,44-46]. Hence, pulses of 1.6, 1.8, and 2.0 kV were administered, corresponding to field strengths of 4.0, 4.5, and 5.0 kV cm^-1^ (Figure 5A), using a capacitance of 25 μF and a resistance of ∞ Ω (i.e., without the use of Pulse Controller module). A voltage of 1.8 kV was found to produce the greatest electrotransformation efficiency, although pulses of 1.6 and 2.0 kV only slightly reduced the electrotransformation efficiency. Pulse duration decreased by approximately 1 ms when increasing pulse voltage from 1.6 to 1.8 kV and from 1.8 to 2.0 kV. Next, capacitances of 25 and 50 μF were assessed at voltages of 1.8 and 2.25 kV, and ∞ Ω (Figure 5B). At both voltages, increasing the capacitance from 25 to 50 μF reduced electrotransformation efficiency by a factor of 2.7 (1.8 kV) and 15.6 (2.25 kV), respectively. Similarly, decreasing resistance from ∞ Ω to 200 and 600 Ω, at 2.25 kV and 25 μF was unproductive and resulted in a 3.3- and 2.3-fold decrease in electrotransformation efficiency, respectively (Figure 5C). Pulse duration changes were not predictive of the effects on electrotransformation efficiency, as increases in the time constant accompanying changes in capacitance and decreases in the time constant accompanying changes in resistance both correlated with decreased electrotransformation efficiency.

(v) **DNA quantity and outgrowth duration.** Finally, we evaluated the effect of DNA amount on both number of transformants and electrotransformation efficiency (Figure 6A) and the effect of the duration of outgrowth following electroporation. Although the total number of transformants was found to increase linearly between 0.5 and 5.0 μg of pMTL85141, the greatest electrotransformation efficiency occurred using 0.5 μg of plasmid DNA. Transformants could be detected at the lowest quantity of DNA tested, 0.25 μg, and saturation with pMTL85141 was not observed up to 5.0 μg, the highest quantity of DNA tested.

For assessing outgrowth duration, we incubated electroporated cells for 0, 2, 4, 6, or 16 hours prior to plating on selective medium. Growth in the form of gas formation and increased culture turbidity could be detected as early as 2 hours following transfer to recovery medium. Transformants could be obtained without recovery (i.e., 0 hours incubation), although at a significantly reduced efficiency (7.9- to 12.1-fold reduction compared to 2–16 hours incubation) (Figure 6B). As expected, the greatest electrotransformation efficiency was attained using the longest recovery time tested (i.e., 16 hours), which was approximately 1.3-fold greater than at 4–6 hours outgrowth, during which time the electrotransformation efficiency was unchanged. While 16 hours of outgrowth is a convenient duration due to the length of the pre-growth and washing and electroporation phases, electrotransformation efficiency for clostridia is typically reported following 4–6 hours of outgrowth. Thus, the electrotransformation efficiencies reported here, all of which involved 16 hour outgrowth experiments, can be divided by 1.3 for comparision to other clostridial electroporation efficiencies.

### Application of the electrotransformation protocol to other vectors

Since many clostridial vectors favor the *ermB* determinant for erythromycin or clarithromycin selection, rather than *catP*-based thiamphenicol selection used in this study, we constructed pMTL85141ermB, a dual *catP* and *ermB* selectable plasmid. Comparable, high-level electrotransformation efficiencies (1.0-1.4 × 10^4^ transformants μg^-1^ DNA) were obtained by selection of pMTL85141ermB using 15 μg/ml thiamphenicol, 4 μg/ml clarithromycin, or 20 μg/ml erythromycin. Control plasmid transformations lacking the *ermB* determinant failed to generate clarithromycin- or erythromycin-resistant colonies. Therefore, *ermB*-based clarithromycin or erythromycin selection is effective using *C. pasteurianum*.

To determine the generality of our high-efficiency electrotransformation protocol for other vectors, we also attempted electrotransfer of pSY6catP into *C. pasteurianum*. pSY6catP is a modified form of pSY6 [47] whereby the *ermB* erythromycin-resistance determinant is replaced with *catP* from pMTL85141. pSY6 is one of several *E. coli*-*Clostridium* shuttle vectors (in addition to, e.g., the ClosTron system of vectors [48]), which harbours the Ll.ltrB group II intron machinery necessary for performing intron-mediated gene knockouts in clostridia. A pSY6-based vector was chosen because it possesses the same pIM13 replicon as pMTL85141, thereby eliminating potential variation in efficiency due to differences in the origin of replication. Unexpectedly, pSY6catP transformed *C. pasteurianum* at a significantly decreased efficiency of 1.1 × 10^1^ transformants μg^-1^ DNA, an efficiency approximately 1,000-fold lower than achieved with pMTL85141. To rule out a vector size effect on the reduction in electrotransformation efficiency (pSY6catP is 8,498 bp, whereas pMTL85141 is 2,963 bp), we also attempted to transform pHT3, a 7,377 bp vector with the same fundamental vector components as pMTL85141ermB, in addition to a heterologous *lacZ* gene from *Thermoanaerobacterium thermosulfurogenes* EM1 [49] (Table 2). Unlike pSY6catP, pHT3 transformed at a high efficiency of 1.8 × 10^4^ transformants μg^-1^ DNA, which is comparable to pMTL85141ermB. Therefore, the dramatic reduction in electrotransformation efficiency is likely not due to differences in plasmid size. At this point, we hypothesize the presence of an additional unidentified restriction system which targets certain common site(s) of pSY6catP, but not pMTL85141, pMTL85141ermB, or pHT3, much like the *dcm*-methylation-dependent restriction systems recently addressed in *C. thermocellum* and *C. ljungdahlii*[27,50]. Our observation of the transformability of *in-vivo-*methylated plasmids, but not *in-vitro-*methylated plasmids, may also be the result of an unidentified methylation-dependent restriction system, which may or may not be the same one affecting pSY6catP. The *in vitro* M.SssI and M.CviPI methyltransferases we utilized, with 5’-^m^CG-3’ and 5’-G^m^C-3’ methylation sites, respectively, each methylate a larger number of sites than the M.FnuDII *in vivo* methyltransferase (5’-^m^CGCG-3’ methylation site). Since certain DNA substrates containing 5-methylcytosine are restricted, it is likely that over-methylated pMTL85141 could be unexpectedly targeted by a Type II or Type IV 5-methylcytosine-specific restriction endonuclease in *C. pasteurianum*. Experiments are underway to probe for an additional restriction system in *C. pasteurianum*. Nonetheless, even with the reduced electrotransformation efficiency of pSY6catP, we have used it to successfully introduce type II introns into the *C. pasteurianum* genome in preliminary experiments (data not shown), whereas this was not possible with the low-level electrotransformation efficiency we initially obtained prior to our electrotransformation studies detailed in this report.

**Table 2:** Strains, plasmids, and oligonucleotides

		
**Strain**	**Relevant characteristics**	**Source or reference**
*Escherichia coli* DH5α	F^-^*endA1 glnV44 thi-1 recA1 relA1 gyrA96 deoR nupG ϕ80dlacZΔM15 Δ(lacZYA-argF)U169, hsdR17(r*_*K*_^*-*^*m*_*K*_^*+*^*), λ*^*-*^	Lab stock
*Escherichia coli* ER1821	F^-^*endA1 glnV44 thi-1 relA1? e14*^*-*^*(mcrA*^*-*^*) rfbD1? spoT1? Δ(mcrC-mrr)114::IS10*	Lab stock; New England Biolabs
*Clostridium pasteurianum* ATCC 6013	Wild-type	American Type Culture Collection
**Plasmid**	**Relevant characteristics**	**Source or reference**
pET-20b(+)	*E. coli* pET-series expression vector (Ap^R^; ColE1 ori)	Novagen
pETKnFRT	Derived by inserting the FRT-flanked kan gene of pKD4 into the MCS of pET-20b(+) (Ap^R^; ColE1 ori; FRT-Kn^R^-FRT)	This study
pFnuDIIM	The M.FnuDII methyltransferase gene of *Fusobacterium nucleatum* inserted into the *tet* gene of pACYC184 (p15A ori; Cm^R^)	[65]
pFnuDIIMKn	Derived by inserting the FRT-flanked *kan* gene of pETKnFRT into the *cat* gene of pFnuDIIM (p15A ori; Kn^R^)	This study
pHT3	*E. coli-C. pasteurianum* shuttle vector containing *lacZ* from *Thermoanaerobacterium thermosulfurogenes* EM1 (Ap^R^; ColE1 ori; Erm^R^; pIM13 ori)	[49]
pIMP1	*E. coli-C. pasteurianum* shuttle vector (Ap^R^; ColE1 ori; Erm^R^; pIM13 ori)	[26]
pKD4	Template vector (Ap^R^; pR6K ori; FRT-Kn^R^-FRT)	[70]
pMTL82151	*E. coli-C. pasteurianum* shuttle vector (Cm^R^; ColE1 ori; pBP1 ori)	[34]
pMTL83151	*E. coli-C. pasteurianum* shuttle vector (Cm^R^; ColE1 ori; pCB102 ori)	[34]
pMTL84151	*E. coli-C. pasteurianum* shuttle vector (Cm^R^; ColE1 ori; pCD6 ori)	[34]
pMTL85141	*E. coli-C. pasteurianum* shuttle vector (Cm^R^; ColE1 ori; pIM13 ori)	[34]
pMTL85141ermB	Derived by insertion of the *ermB* gene of pIMP1 into pMTL85141	This study
pSC12	*E. coli-C. pasteurianum* shuttle vector (Cm^R^; ColE1 ori; pIM13 ori)	[66]
pSY6	*E. coli-C. pasteurianum* expression vector carrying the *L. lactis ltrB* group II intron under control of the *C. acetobutylicum ptb* promoter, and *ltrA* ORF (Ap^R^; ColE1 ori; Erm^R^; pIM13 ori)	[47]
pSY6catP	Derived by replacing the *ermB* gene of pSY6 with the *catP* gene from pSC12	This study
**Oligonucleotide**	Sequence (5’-3’)^*^	
KnFRT.BlpI.S	ACACGTGCTCAGCGATTGTGTAGGCTGGAGCTGCTTCG	
KnFRT.XhoI.AS	GCCATGCTCGAGATGAATATCCTCCTTAGTTCCTATTCC	
ermB.NdeI.S	ATTACGCATATGTTTGGCTAACACACACGCCATTCC	
ermB.PvuI.AS	CTTTTTCGATCGTTTCCGACGCTTATTCGCTTCGCT	
catP.BclI.S	GTTTGATCATGGTCTTTGTACTAACCTGTGG	
pSC12.SOE.AS	tacagcatgaccgttaaagtgg	
pSC12.SOE.S	ccactttaacggtcatgctgtaAGTGCAAGGTACACTTGCAAAGTAGTGG	
catP.ClaI.AS	GGATCGATCCAACTTAATCGCCTTGCAGCACA	
pMTL.seq.S	GGGAGGTCAATCTATGAAATGCG	
pMTL.seq.AS	CGGAGCATTTGGCTTTCCTTCCAT	

* Lower case: overlap sequences used in SOE PCR; Underline: restriction recognition sequences.

## Discussion

Lack of a gene transfer system has fundamentally hindered genetic manipulation of *C. pasteurianum*. We report here the first development of an electroporation-mediated procedure for high-level gene transfer to *C. pasteurianum*. The first step in overcoming this barrier involved confirming the previously identified *C. pasteurianum* restriction endonuclease, CpaAI, which cleaves 5’-CGCG-3’ nucleotide sequences. *In vivo* methylation using the M.FnuDII methyltransferase allowed protection of *E. coli*-*C. pasteurianum* shuttle vectors. Active protoplast extracts of *C. pasteurianum* completely degraded pMTL85141 prepared from *E. coli* DH5α, but not when prepared from *E. coli* ER1821 harboring both pMTL85141 and pFnuDIIMKn (Figure 1A, B). This finding allowed us to demonstrate low-level electrotransformation of *C. pasteurianum* by using conditions commonly utilized for clostridial electroporation (Table 1). Despite repeated attempts, unmethylated pMTL85141 (i.e., prepared from *E. coli* DH5α in the absence of plasmid pFnuDIIMKn) was unable to transform *C. pasteurianum*, confirming the requirement for proper methylation of plasmid DNA prior to electrotransformation. Plasmid transfer was confirmed through colony-PCR-amplification, isolation and purification, and DNA sequencing of plasmid pMTL85141 from thiamphenicol-resistant colonies (Figure 2A, B; data not shown). The resulting plasmid preparation from *C. pasteurianum* was devoid of rearrangements and recombination and could only transform restriction-deficient strains of *E. coli*, such as ER1821. The initial efficiency of approximately 2.4 × 10^1^ transformants μg^-1^ DNA paled in comparison to electrotransformation levels achieved with other species of *Clostridium* (up to 10^6^ transformants μg^-1^ DNA [35]) and prevented transfer of low-level-transforming vectors, such as pSY6catP (discussed below). Thus, we investigated the effects of several parameters critical for the electrotransformation of Gram-positive bacteria. With the modified protocol, we were able to elevate the electrotransformation efficiency to a maximum of 7.5 × 10^4^ transformants μg^-1^ DNA, an increase of more than three orders of magnitude from the original low-level electrotransformation efficiency (Table 3). The electroporation parameters determined to be most influential on the electrotransformation of *C. pasteurianum* are discussed in detail below.

**Table 3:** **Summary of protocol for high-level electrotransformation of *C. pasteurianum *and comparison to initial low-level protocol**

**Electrotransformation parameter**	**Low-level protocol**	**High-level protocol**
*Cell growth*		
Growth additive	None	1.25% glycine (at OD_600_ 0.3-0.4)
Osmotic stabilizer	None	Hypertonic sucrose (0.4 M; at OD_600_ 0.3-0.4)
OD_600_ at time of harvest	OD_600_ 0.6-0.8	OD_600_ 0.6-0.8
*Washing and pulse delivery*		
Osmotic stabilizer	Isotonic sucrose (0.27 M)	Isotonic sucrose (0.27 M)
Cell membrane solubilizer	None	5% (v/v) ethanol 5 min prior to pulse
DNA amount	5 μg	0.5 μg
Pulse parameters	2.0-2.5 kV; 25 μF; 200–800 Ω; 6–9 ms	1.8 kV; 25 μF; ∞ Ω; 12–14 ms
*Outgrowth*		
Osmotic stabilizer	None	Hypotonic sucrose (0.2 M)
Recovery time	16 h	4-6 h
Transformation efficiency	2.4 × 10^1^ transformants μg^-1^ pMTL85141	Up to 7.5 × 10^4^ transformants μg^-1^ pMTL85141

Gram-positive bacteria are electrotransformed at efficiencies several orders of magnitude lower than Gram-negative cells, highlighting the importance of effective weakening of the Gram-positive cell wall prior to electroporation. The most widely used additives for this purpose are glycine, dl-threonine, lysozyme, and penicillin G, all of which were screened in this study (Figure 3A). Under the conditions tested, only glycine and dl-threonine afforded enhanced levels of electrotransformation, with glycine rendering the cell most susceptible to electrotransformation. Glycine has been widely used for increasing electrotransformation efficiency of Gram-positive bacteria [36,37,39,51-56] and its mode of action has been extensively studied [57]. Specifically, glycine is incorporated into the peptidoglycan network through replacement of critical d- and l-alanine residues, generating a weakened cell wall due to a lesser degree of cross-linking [57]. To balance glycine supplementation with cell viability, we determined that optimum electroporation is achieved using brief exposure to the highest glycine concentration that still permits sufficient cell growth. The optimized glycine regimen for *C. pasteurianum* involves exposure of early exponential phase cells to 1.25% glycine for 2–3 hours (Figure 3B, C), which is most similar to protocols developed for *Mycobacterium avium*[37], *Bacillus subtilis*[54], and *B. thuringiensis*[55]. Of particular importance is the timing of glycine addition. Specifically, glycine should be added only when the cells enter the exponential growth phase, as glycine supplementation at the time of inoculation resulted in significant growth inhibition. While cell-wall-weakening is not an indispensable requirement for electrotransformation of *C. pasteurianum*, a significant increase in electrotransformation efficiency was obtained with glycine treatment. To our knowledge this study represents the first use of glycine as a cell-wall-weakening and electroporation-enhancing agent within the *Clostridium* genus.

Upon treatment with glycine, cells possess a compromised cell wall, and therefore, it is paramount that cells be stabilized osmotically. Our results clearly demonstrate the importance of sucrose osmoprotection during glycine treatment, as cultures without sucrose supplementation failed to grow in the presence of glycine. Although 0.25 M sucrose in the growth medium was sufficient to promote growth, increasing the concentration to 0.4 M afforded a significant enhancement to electrotransformation (Figure 3B). In opposition to the growth medium, however, hypertonic sucrose in the recovery medium (0.4 M) was found to have a slightly negative effect on electrotransformation efficiency compared to a slightly hypotonic environment (0.2 M) (Figure 3E). A greater degree of osmotic stabilization is likely required in the growth medium compared to the recovery medium as a result of cell-wall-weakening that occurs in the presence of glycine. In a similar manner, hypertonic sucrose (0.5 M) in the wash and electroporation buffer greatly reduced electrotransformation efficiency compared to an isotonic buffer (0.27 M) (Figure 3D). This outcome is in agreement with other clostridial electrotransformation protocols that employed phosphate-buffered sucrose as the wash and electroporation buffer, as all such protocols utilize isotonic rather than hypertonic sucrose [25,26,44-46,58,59]. Alternatively, a hypertonic wash and electroporation buffer has been shown to enhance electrotransformation of *Streptococcus cremoris* and *S. lactis*[60], *Listeria monocytogenes*[61], and *Lactobacillus sake*[62]. Based on these studies and our data presented here it is evident that the degree of osmotic protection required to achieve optimum electrotransformation must be carefully determined for each phase of the electroporation process. While it is commonly assumed that the role of osmotic stabilizer on cell-wall-weakened and electrotransformed cells is strictly protective, it cannot be ruled out that higher electrotransformation efficiencies arise, at least in part, from faster growth rates exhibited by cultures that actively utilize the stabilizer as a carbon and energy source, as observed with the use of sucrose for *C. pasteurianum* (this study) and *Lactococcus lactis* subsp. c*remoris*[36].

In addition to the use of cell-wall-weakening additives, the Gram-positive cell wall can be destabilized through the application of high-voltage pulses during electroporation, as stronger electric fields are typically required for Gram-positive compared to Gram-negative bacteria [23,24]. Hence, optimization of the electrical parameters must be performed to achieve efficient electroporation without compromising cell viability. We aimed to tailor our electric pulse, defined by the pulse voltage (kV) and corresponding field strength (kV cm^-1^), capacitance (μF), and resistance (Ω), based on our developed glycine and sucrose regimen. In contrast to most clostridial electroporation procedures, which commonly utilize voltages of 2.0-2.5 kV (5.0-6.25 kV cm^-1^), optimum electrotransformation of glycine-treated *C. pasteurianum* was found to occur under a lower voltage of 1.8 kV (4.5 kV cm^-1^; Figure 5A). Within the clostridia, this low field strength has only been matched by the described protocol for *C. acetobutylicum* DSM 792 [45]. Increasing the capacitance at voltages of either 1.8 or 2.25 kV generated significantly reduced electrotranformation efficiencies (Figure 5B). In addition, using a higher voltage of 2.25 kV and attempting to truncate the duration of the pulse by using lower resistances of 200 and 600 Ω also produced a reduced number of transformants (Figure 5C). Therefore, optimum electrotransformation of glycine-treated *C. pasteurianum* was found to occur under a relatively low electric field of 4.5 kV cm^-1^ at 25 μF and ∞ Ω, generating a time constant of 12–14 ms. We postulate that a relatively weak electric field is best for enhancing electroporation of *C. pasteurianum* as a result of the compromised cell wall associated with the application of our glycine regimen. A similar effect has been observed using glycine-treated cells of *Bacillus cereus*, in which glycine had no effect under a high field strength (20 kV cm^-1^), yet a pronounced positive effect under a low field strength (12 kV cm^-1^) [56].

To a lesser extent than the Gram-positive cell wall, the cell membrane also acts as a physical barrier to transfer of plasmid DNA into the cell. Aside from the presumed pore formation that occurs immediately following delivery of the electric pulse, little work has been done to enhance electroporation through increasing the extent of membrane permeabilization. Recently, two reports have detailed the use of ethanol as a membrane-solubilizing agent to enhance electroporation of *Escherichia coli*[41] and *Oenococcus oeni*[40]. The effect of ethanol, a fermentation end product of *C. pasteurianum*, on growth of species of *Clostridium* has been extensively studied and it has been shown that ethanol toxicity occurs through direct interaction with the cell membrane resulting in a decreased extent of lipid organization and increased membrane fluidity and cytoplasmic leakage [42,63]. It has been proposed that leakage occurs through an ethanol-induced increase in membrane pore size [64], which has clear implications to electroporation. In this report we assessed the electroporation-enhancing effect of ethanol and also extended our approach to butanol. Whereas butanol inhibited electrotransformation and cell growth of *C. pasteurianum* under the conditions tested, we found that ethanol supplemented at an appropriate concentration (5 or 10%) had a clear positive effect on electrotransformation (Figure 4). To our knowledge, this study represents the first use of ethanol to enhance electrotransformation within the *Clostridium* genus.

The investigation of various electrotransformation parameters enabled us to generate a maximum of 7.5 × 10^4^ transformants μg^-1^ DNA, a more than 3,000-fold increase compared to our initial attempts using common clostridial electroporation conditions (Table 3). The results reported in this study demonstrate that *C. pasteurianum* ATCC 6013 is amendable to genetic manipulation. Our hope is that our developed gene transfer protocol will allow genetic and metabolic engineering of *C. pasteurianum* and promote further development of this biotechnologically important microorganism, as the maximum electrotransformation efficiency attained for *C. pasteurianum* is among the highest reported in the *Clostridium* genus. However, significant barriers remain to be resolved; namely the low electrotransformation efficiency for plasmids which carry group II intron machinery, such as pSY6catP, necessary for constructing gene knockout mutants in clostridia. Since we have observed a drop in electrotransformation efficiency for pSY6catP (8,498 bp), yet not pHT3 (7,377 bp), a plasmid of comparable size, we suspect the decrease in efficiency is not related to plasmid size. Instead, we speculate that *C. pasteurianum* possesses at least one additional restriction-modification system, in addition to CpaAI, that is specifically active on pSY6catP, but not on pMTL85141. It is likely that additional uncharacterized restriction activities are also responsible for our inability to electrotransform substrates methylated *in vitro* using CpG and GpC methyltransferases, despite proper protection against the previously identified CpaAI restriction endonuclease. We are currently conducting genome sequencing of *C. pasteurianum* which will enable us to identify potential candidate restriction-modification genes that may be responsible for the low electrotransformation efficiency of certain non-pMTL85141 and *in-vitro-*methylated vectors. Also, it should be mentioned that, even with the reduced electrotransformation efficiency of pSY6catP, our electrotransformation protocol developed herein allows the introduction of group II introns into the *C. pasteurianum* genome in preliminary experiments (data not shown). Thus, should genome sequencing reveal additional restriction-modification genes, our electrotransformation method should enable investigators to knockout such genes using group II introns. Finally, the work here also demonstrates that *catP* (using thiamphenicol) and *ermB* (using clarithromycin or erythromycin) comprise effective selection marker systems for performing genetic engineering in *C. pasteurianum*. The pIM13 origin of replication from *Bacillus subtilis* and the pCB102 and pCD6 origins of replication from *C. butyricum* and *C. difficile*, respectively, were shown to support plasmid maintenance in *C. pasteurianum* and they round out the vector toolkit now available for genetic engineering in this important bacterium. Taken together, the high-level electrotransformation protocol and the vector and selection tools described herein set the stage for biotechnological exploitation of *C. pasteurianum* for the first time, thereby opening an important avenue for the production of biofuels from low-value and abundant crude glycerol.

## Conclusions

In this work, M.FnuDII methylation, together with cell-wall-weakening, partial membrane solubilization, a low electric field, and osmoprotection enabled the electrotransformation of *C. pasteurianum* ATCC 6013 at an efficiency of up to 7.5 × 10^4^ transformants ug^-1^ DNA. The work here also demonstrates the development of a *C. pasteurianum* genetic toolkit currently comprised of two selectable markers (*catP*-based thiamphenicol selection and *ermB*-based clarithromycin or erythromycin selection) and three Gram-positive origins of replication (pIM13 from *Bacillus subtilis*, pCD6 from *C. difficile*, and pCB102 from *C. butyricum*). This is the first report of a genetic transformation procedure for *C. pasteurianum* and represents a key advancement for this industrially-important bacterium with important implications for low-cost biofuel production.

## Methods

### Bacterial strains, plasmids and primers

The bacterial strains, plasmids, and oligonucleotides utilized in this work are listed in Table 2. *E. coli* DH5α was utilized for routine vector construction and propagation, and *E. coli* ER1821 for maintenance of M.FnuDII-methylated *E. coli*-*C. pasteurianum* shuttle vectors. *C. pasteurianum* ATCC™ 6013 (Winogradsky 5; W5) was acquired from the American Type Culture Collection (Manassas, VA, USA). Modular pMTL-series shuttle vectors [34] were kindly provided by Prof. Nigel Minton (University of Nottingham, Nottingham, UK). Plasmids pFnuDIIM [65], pSC12 [66], and pSY6 [47] were respectively provided by Dr. Geoffrey Wilson (New England Biolabs, Inc. (NEB), Ipswich, MA, USA), Prof. George Bennett (Rice University, Houston, TX, USA), and Prof. Sheng Yang (Shanghai Institutes for Biological Sciences, Shanghai, China). Plasmids pHT3 [49] and pIMP1 [26] were provided by Prof. Terry Papoutsakis (University of Delaware, Newark, DE, USA). Oligonucleotide primers were synthesized and purified by Integrated DNA Technologies (IDT; Iowa City, IA, USA) using standard desalting.

### Bacterial growth and maintenance conditions

Unless stated otherwise, all chemicals were purchased from Sigma-Aldrich (St. Louis, MO, USA) and stock solutions were prepared according to the manufacturer’s recommendations. *E. coli* strains were grown aerobically at 37°C in lysogeny broth (LB; 10 g/l NaCl, 5 g/l Bacto yeast extract, and 10 g/l Bacto tryptone). Solid and liquid cultures of recombinant *E. coli* were supplemented with 100, 34, or 30 μg/ml of ampicillin, chloramphenicol, and kanamycin, respectively. For selection of strains harboring two compatible plasmids, antibiotic concentrations were reduced by 50%. Recombinant *E. coli* stocks were stored at −80°C in 15% glycerol. Unless specified otherwise, growth and manipulation of *C. pasteurianum* was performed in a controlled anaerobic atmosphere (85% N_2_, 10% H_2_, and 5% CO_2_) within an anaerobic chamber (Plas-Labs, Inc.; Lansing, MI, USA). Oxygen was purged from growth medium by autoclaving and trace O_2_ was reduced using a palladium catalyst fixed to the heating unit of the anaerobic chamber. Agar-solidified medium was prepared aerobically and allowed to equilibrate within the anaerobic chamber for at least 36 hours prior to use. Anaerobic conditions were monitored by addition of 1 mg/l resazurin to both solid and liquid media. Solid and liquid cultures of recombinant *C. pasteurianum* were supplemented with 15 μg/ml thiamphenicol. Cells were maintained as spores on solidified 2×YTG (16 g/l Bacto tryptone, 10 g/l Bacto yeast extract, 5 g/l glucose, 5 g/l NaCl, and 12 g/l agar) plates. Sporulated agar plate stocks were prepared by streaking colonies from an exponential-phase culture (OD_600_ of 0.4-0.6) and cultivating for more than seven days under anaerobic conditions, followed by exposure and storage in air at 4°C for up to two months [67]. For long-term storage, vegetative stock cultures (OD_600_ of 0.4-0.6) were prepared and stored at −80°C in 10% glycerol by inoculating a single sporulated plate colony into 10 ml 2×YTG and heat shocking at 80°C for 10 minutes to induce germination.

### Preparation of protoplasts and assay of CpaAI activity

Protoplasts of *C. pasteurianum* were prepared by suspension of cells from a 100 ml culture (OD_600_ of 0.4-0.6) in 25 ml of protoplast buffer (25 mM potassium phosphate, pH 7.0, 6 mM MgSO_4_, and 15% lactose) containing 200 μg/ml lysozyme, followed by incubation for 45 minutes anaerobically at 37°C, as described previously [19,68]. For preparation of crude protoplast lysates, 25 ml of protoplasts were collected by centrifugation at 8,500×g for 20 minutes and lysed by resuspension in 20 ml of TEMK buffer (4 mM Tris–HCl, pH 8.0, 10 mM EDTA, 6.6 mM 2-mercaptoethanol, and 25 mM KCl) [26]. After incubation at 37°C for 1 hour, cell debris was cleared by centrifugation at 20,000×g for 15 minutes and supernatants containing protoplast extracts were stored at −80°C. CpaAI activity was assayed as previously described [20]. Reaction mixtures contained 1.0 μg plasmid DNA and 25% crude protoplast lysate in a total volume of 20 μl of 1× CpaAI reaction buffer (6 mM Tris–HCl, pH 7.4, 6 mM MgCl_2_, and 6 mM 2-mercaptoethanol). Optimal digestion occurred at 37°C for 2–4 hours.

### DNA Isolation and manipulation

Plasmid DNA was extracted and purified from *E. coli* DH5α and ER1821 using an EZ-10 Spin Column Plasmid DNA Miniprep Kit from Bio Basic, Inc. (Markham, ON, Canada). Recombinant DNA manipulations were performed according to standard procedures [69]. *Taq* DNA polymerase, restriction endonucleases, CpG (M.SssI) and GpC (M.CviPI) methyltransferases, Quick Ligation Kit, and 1 kb DNA ladder were purchased from NEB (Ipswich, MA, USA). *Pfu* DNA polymerase and RNase A were purchased from Bio Basic, Inc. (Markham, ON, Canada). All commercial enzymes and kits were used according to the manufacturer’s instructions.

Plasmid DNA was extracted and purified from *C. pasteurianum* using a previously described method [49]. Briefly, 3–9 ml of late-exponential phase cells were collected by centrifugation and washed twice in KET buffer (0.5 M KCl, 0.1 M EDTA, and 0.05 M Tris–HCl, pH 8.0) and once in SET buffer (25% sucrose, 0.05 M EDTA, and 0.05 M Tris–HCl, pH 8.0). Cells were then suspended in 200 μl of SET buffer containing 5 mg/ml lysozyme and incubated anaerobically at 37°C for 20 minutes. RNase A was added to a final concentration of 100 μg/ml and cell lysis and plasmid purification were carried out using the protocol for Purification of Low-Copy Number Plasmid and an EZ-10 Spin Column Plasmid DNA Miniprep Kit from Bio Basic, Inc. (Markham, ON, Canada) beginning with addition of 400 μl of alkaline SDS solution II.

Colony PCR of wild-type and recombinant *C. pasteurianum* was performed by suspending single colonies in 50 μl colony lysis buffer (20 mM Tris–HCl, pH 8.0, containing 2 mM EDTA and 1% Triton X-100), heating in a microwave for 2 minutes at maximum power setting, and adding 1 μl of the resulting cell suspension to a 9 μl PCR containing Standard Taq DNA Polymerase (NEB; Ipswich, MA, USA). An initial denaturation of 5 minutes at 95°C was employed to further cell lysis. Colonies screened in this manner by suspension in deionized H_2_O failed to yield appreciable amplification.

### Vector construction

Plasmid pFnuDIIMKn was derived from pFnuDIIM to allow methylation of *E. coli-C. pasteurianum* shuttle vectors and possesses a kanamycin-resistance determinant, as both pFnuDIIM [65] and the *E. coli-C. pasteurianum* shuttle vectors used in this study carry the same chloramphenicol-resistance marker. First, an FRT-*kan*-FRT PCR cassette was amplified from plasmid pKD4 [70] using primers KnFRT.BlpI.S and KnFRT.XhoI.AS and inserted into the MCS of BlpI/XhoI-digested pET-20b(+) (Novagen; Madison, WI, USA) to generate pETKnFRT. Next, the FRT-*kan*-FRT cassette was digested out of pETKnFRT using ScaI and EcoRI and subcloned into the corresponding restriction sites within the *catP* gene of pFnuDIIM to yield pFnuDIIMKn.

Plasmid pSY6catP was derived from pSY6 [47] by swapping the *ermB* marker with the *catP* determinant from pSC12 [66]. The internal BsrGI recognition site within the coding sequence of *catP* was mutated by introducing two silent mutations using splicing by overlap extension (SOE) PCR to prevent interference with future group II intron retargeting, which requires use of BsrGI. The *catP* gene was amplified in two parts from template pSC12 using primer sets catP.BclI.S/pSC12.SOE.AS and pSC12.SOE.S/catP.ClaI.AS with 22 bp of overlap between products. The resulting overlapping PCR products were separated on a 2.0% agarose gel, pierced three times with a P10 micropipette tip, and used as template in a SOE PCR by cycling for 10 cycles prior to adding primers catP.BclI.S and catP.ClaI.As and cycling for 25 additional cycles. The mutated PCR product was purified using a EZ-10 Spin Column PCR Products Purification Kit (Bio Basic, Markham, ON, Canada), digested with BclI/ClaI, and inserted into the corresponding sites of pSY6 to generate pSY6catP.

Plasmid pMTL85141ermB was derived from pMTL85141 via insertion of the *ermB* marker from pIMP1 into pMTL85141. The *ermB* gene and associated promoter was PCR-amplified from template pIMP1 using primers ermB.NdeI.S and ermB.PvuI.AS. The resulting 1,238 bp PCR product was purified using an EZ-10 Spin Column PCR Products Purification Kit (Bio Basic, Markham, ON, Canada), digested with NdeI/PvuI, and inserted into the corresponding sites of pMTL85141 to generate pMTL85141ermB.

### Preparation of electrocompetent cells and electrotransformation

For preparation of electrocompetent cells of *C. pasteurianum* using the high-level protocol, a seed culture was first prepared by inoculating 20 ml of reduced 2×YTG with 0.2 ml of a thawed glycerol stock. The culture was then 20^-2^-diluted and, following overnight growth at 37°C, 1 ml of the seed culture was transferred to a 125 ml Erlenmeyer flask containing 20 ml of reduced 2×YTG. Cells were grown to early exponential phase (OD_600_ of 0.3-0.4), at which time filter-sterilized stock solutions of 2 M sucrose and 18.77% glycine were added to respective concentrations of 0.4 M and 1.25%. Growth was resumed until the culture attained an OD_600_ of 0.6-0.8 (approximately 2–3 h) and 20 ml culture was transferred to a 50 ml pre-chilled, screw-cap centrifuge tube. At this point, all manipulations were performed at 4°C using an ice-bath and pre-chilled reagents. Cells were removed from the anaerobic chamber and collected by centrifugation at 8,500×g and 4°C for 20 minutes. The resulting cell pellet was returned to the anaerobic chamber and washed once in 5 ml of filter-sterilized SMP buffer (270 mM sucrose, 1 mM MgCl_2_, and 5 mM sodium phosphate, pH 6.5). Following centrifugation, the final cell pellet was resuspended in 0.6 ml SMP buffer.

For transfer of plasmids to *C. pasteurianum*, *E. coli-C. pasteurianum* shuttle vectors were first co-transformed with pFnuDIIMKn into *E. coli* ER1821 to methylate the external cytosine residue within 5’-CGCG-3’ tetranucleotide recognition sites of CpaAI. Plasmid mixtures were then isolated and 0.5 μg, suspended in 20 μl of 2 mM Tris–HCl, pH 8.0, was added to 580 μl of *C. pasteurianum* electrocompetent cells. The cell-DNA mixture was transferred to a pre-chilled electroporation cuvette with 0.4 cm gap (Bio-Rad; Richmond, CA, USA), 30 μl of cold 96% ethanol was added, and the suspension was incubated on ice for 5 minutes. A single exponential decay pulse was applied using a Gene Pulser (Bio-Rad, Richmond, CA, USA) set at 1.8 kV, 25 μF, and ∞ Ω, generating a time constant of 12–14 ms. Immediately following pulse delivery, the cuvette was flooded with 1 ml 2×YTG medium containing 0.2 M sucrose and the entire suspension was transferred to 9 ml of the same medium. Recovery cultures were incubated for 4–6 hours prior to plating 50–250 μl aliquots onto 2×YTG agar plates containing 15 μg/ml thiamphenicol, 4 μg/ml clarithromycin, or 20 μg/ml erythromycin. Plates were incubated for 2–4 days under secondary containment within 3.4 L Anaerobic Jars each equipped with a 3.5 L Anaerobic Gas Generating sachet (Oxoid Thermo Fisher; Nepean, ON, Canada).

## Abbreviations

BuOH: butanol; DNA: deoxyribonucleic acid; ET: electrotransformaion; EtOH: ethanol; FRT: flippase recognition target; Gly: glycine; Ll.ltrB: mobile group II intron from *Lactococcus lactis*; MCS: multiple cloning site; NR: non-recombinant; PCR: polymerase chain reaction; SMP: sucrose-magnesium-phosphate (buffer); SOE PCR: splicing by overlap extension polymerase chain reaction; Suc: sucrose; Thr: threonine; Tm: thiamphenicol

## Competing interests

DAC is a founder and employee of Centurion Biofuels Corporation, at which MEP has also been employed. Centurion Biofuels Corporation has a financial interest in production of biofuels using clostridial microorganisms.

## Authors’ contributions

MEP helped conceive of the study, participated in its design and coordination, carried out the transformations, and drafted the manuscript. MMY participated in the study design and coordination. DAC and CPC helped conceive of the study, participated in its design and coordination, and helped to draft the manuscript. All authors read and approved the final manuscript.
